# Revisiting an IgG Fc Loss-of-Function Experiment: the Role of Complement in HIV Broadly Neutralizing Antibody b12 Activity

**DOI:** 10.1128/mBio.01743-21

**Published:** 2021-10-12

**Authors:** Benjamin S. Goldberg, Chengzi I. Kaku, Jérémy Dufloo, Timothée Bruel, Olivier Schwartz, David A. Spencer, Ann J. Hessell, Margaret E. Ackerman

**Affiliations:** a Thayer School of Engineering, Dartmouth College, Hanover, New Hampshire, USA; b Geisel School of Medicine, Dartmouth College, Hanover, New Hampshire, USA; c Division of Pathobiology and Immunology, Oregon National Primate Research Center, Oregon Health & Science University, Beaverton, Oregon, USA; d Virus and Immunity Unit, Department of Virology, Institut Pasteur, Paris, France; The Rockefeller University; Albert Einstein College of Medicine

**Keywords:** antibody, Fc, complement, CDC, viral lysis, C1q, antibody-mediated prevention, mechanism of action, HIV, human immunodeficiency virus

## Abstract

The role of the complement system in HIV-1 immunity and pathogenesis is multifaceted, and an improved understanding of complement activities mediated by HIV-1-specific antibodies has the potential to inform and advance clinical development efforts. A seminal nonhuman primate challenge experiment suggested that complement was dispensable for the protective effect of the early broadly neutralizing antibody (bnAb) b12, but recent experiments have raised questions about the breadth of circumstances under which this conclusion may hold. Here, we reassess the original observation using Fc variants of IgG1 b12 that enhance complement activity and report that complement fixation on recombinant antigen, virions, and cells and complement-dependent viral and cellular lysis *in vitro* vary among bnAbs. Specifically, while the clinically significant V3 glycan-specific bnAb 10-1074 demonstrates activity, we found that b12 does not meaningfully activate the classical complement cascade. Consistent with avid engagement by C1q and its complex system of regulatory factors, these results suggest that complement-mediated antibody activities demonstrate a high degree of context dependence and motivate revisiting the role of complement in antibody-mediated prevention of HIV-1 infection by next-generation bnAbs in new translational studies in animal models.

## INTRODUCTION

Recombinant antibodies represent a proven antiviral intervention (1), with U.S. FDA approval of palivizumab (Synagis) for respiratory syncytial virus (RSV) prophylaxis in infants and the antibody cocktail atoltivimab/maftivimab/odesivimab (Inmazeb) for Ebola virus postexposure prophylaxis and FDA emergency use authorizations granted for three antibody treatments for severe acute respiratory syndrome coronavirus 2 (SARS-CoV-2) infection at the time of writing (2–4). In the absence of an effective vaccine, anti-human immunodeficiency virus type 1 (HIV-1) antibody discovery and clinical development strategies have focused on those that potently neutralize virus via the recognition of epitopes conserved across viral phylogenetic diversity (5). As of May 2020, 28 such broadly neutralizing anti-HIV-1 antibodies (bnAbs) were the subject of 167 clinical trials, of which 32 trials of 9 bnAbs alone or in combination were investigating protection against infection in healthy, uninfected subjects (6).

The highly anticipated results of the first of these studies to report on efficacy, the phase 2b Antibody Mediated Prevention (AMP) trials (HVTN 704/HPTN 085 and HVTN 703/HPTN 081), revealed the bnAb VRC01 to be protective against the acquisition of VRC01 neutralization-sensitive HIV-1 strains (7). The results suggest that antibody prophylaxis is possible in humans, but the risk reduction is more modest than hoped, leaving open the potential of bioengineering strategies to optimize antibody-mediated prevention.

The ability of some antibodies to oligomerize on antigenic surfaces (8) and activate the complement cascade represents an arm of extraneutralizing functions that is well studied and whose importance is well understood in the field of immune oncology (9), yet enthusiasm about its contribution to antibody-mediated protection against HIV-1 infection as a means of augmenting neutralizing antibody drugs was largely extinguished by a seminal nonhuman primate (NHP) passive immunization and challenge experiment designed to determine antibody mechanism of action (10). In this influential study, a variant of the bnAb b12 with Fc domain mutations designed to eliminate Fc receptor (FcγR) and complement initiator C1q binding (LALA) provided protection diminished from that of the unmodified antibody, while a C1q-only knockout (KA) variant demonstrated protection equivalent to that of unmodified b12, suggesting that FcγR- but not complement-mediated functions contributed to protection from infection *in vivo* (10). In contrast, two similar experiments conducted with a more potent bnAb, PGT121, reported no difference in resistance to infection when this antibody was modified to eliminate both FcγR and complement activities (11, 12). Analysis of how the passive transfer of other bnAb-based interventions in the context of established infection has further supported the role of effector functions with *in vivo* antiviral activity (13) but has not defined the role that the complement cascade might play.

Here, we revisit the biological activity of the b12 antibody *in vitro* in an effort to more fully examine the interpretation of the original NHP challenge experiment in the context of ongoing clinical evaluation of antibody-based prophylaxes. Our findings suggest that the conclusion of Hessell et al. (10), that complement does not contribute to b12 activity *in vivo*, is clear and well supported; however, reconsideration of classical complement activity as a factor in either protection or enhanced pathogenesis is warranted for other bnAbs in development. In either scenario, an advanced understanding of the interplay between classical complement and HIV-1 can be productively incorporated into future antibody-mediated prevention strategies.

## RESULTS

### Characterization of a panel of b12 variants.

**FIG 1:**
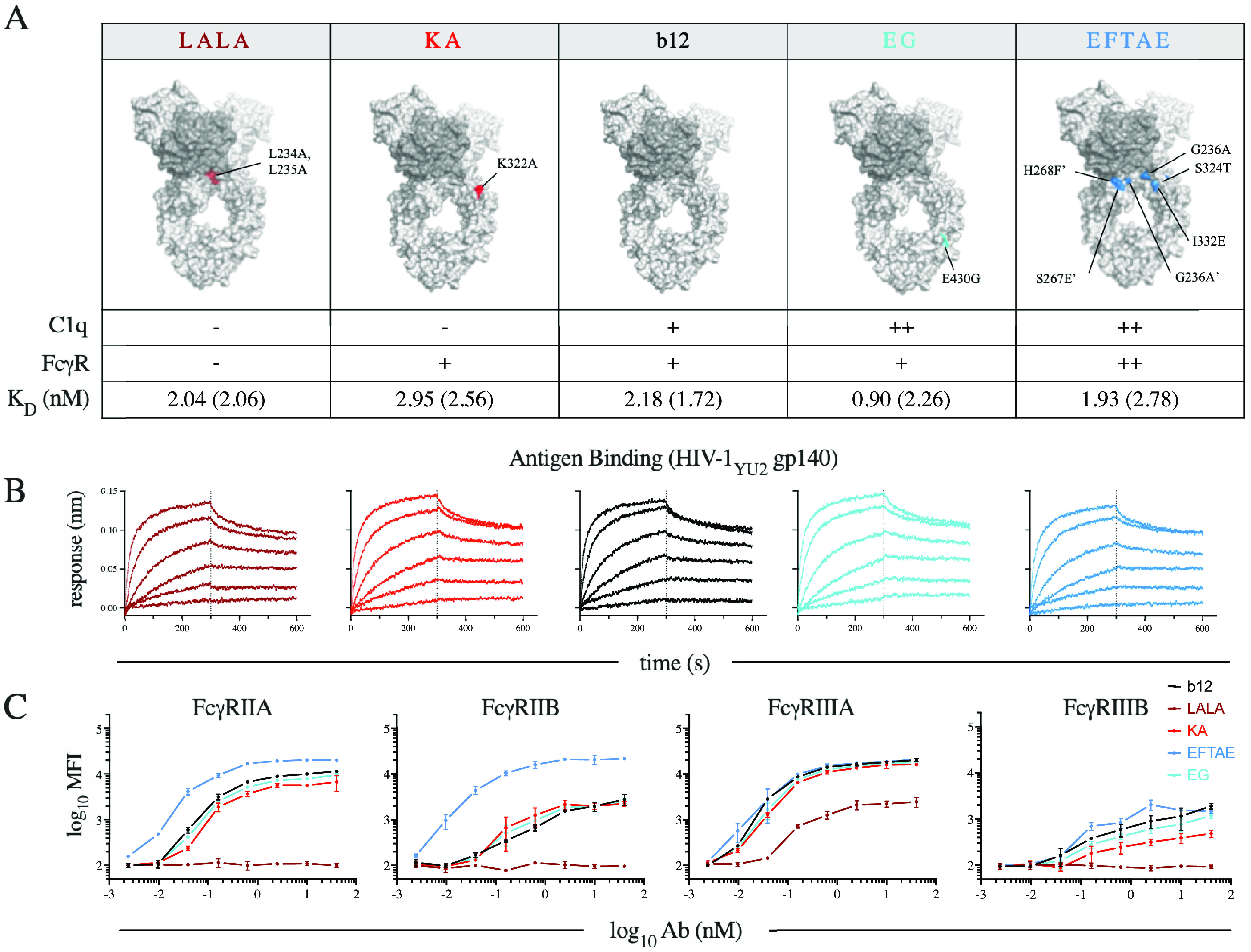
b12 variant panel. (A) Antibody (Ab) IgG1 Fc variants illustrated on the crystal structure of the broadly neutralizing antibody b12 (PDB accession number 1HZH) by coloration of the component point mutations. The phenotypically diminished variants to the left of b12 are illustrated in red (e.g., LALA and KA), while phenotypically enhanced variants to the right are in blue (e.g., EG and EFTAE). Accompanying the illustrations is a table of expected qualitative C1q and FcγR binding phenotypes and observed affinity of antigen binding (equilibrium [kinetic] dissociation constant [*K_D_*] values) of each variant to antigen (HIV-1_YU-2_ gp140 trimer). (B) Antigen binding profiles determined by biolayer interferometry (BLI) across a range of concentrations. (C) FcγR binding profiles of each variant as determined by staining antigen-conjugated beads with tetramerized receptor. Error bars represent the ranges from two technical replicates.

A panel of b12 Fc variants, including those used in the NHP challenge experiment, was designed to assess various *in vitro* measures of complement-dependent antibody activity. The panel consisted of two variants, KA and EG, that are known to possess a selectively modified C1q binding phenotype, and two variants, LALA and EFTAE, that exhibit an altered interaction profile with C1q and across FcγRs (Fig. 1A). The binding knockout variants KA and LALA (14, 15) as well as two enhancing variants, EG (16) and EFTAE (17, 18), were cloned, expressed, and characterized for conformance to literature phenotypes. These antibodies exhibited essentially identical binding affinities for YU-2 gp140 trimers as assessed by biolayer interferometry (BLI) (Fig. 1B) but showed marked differences in their abilities to interact with human FcγRs, as assessed using a customized multiplex assay (Fig. 1C). As others have reported (19), the LALA variant retained moderate binding to tetramerized FcγRIIIA despite being originally characterized as a pan-FcγR knockout (15). Both the complement-enhancing EG variant and the complement knockout KA variant exhibited comparable profiles of FcγR binding to unmodified b12, whereas the dual-complement and FcγR-binding-enhanced EFTAE variant showed elevated binding to FcγR.

**FIG 2:**
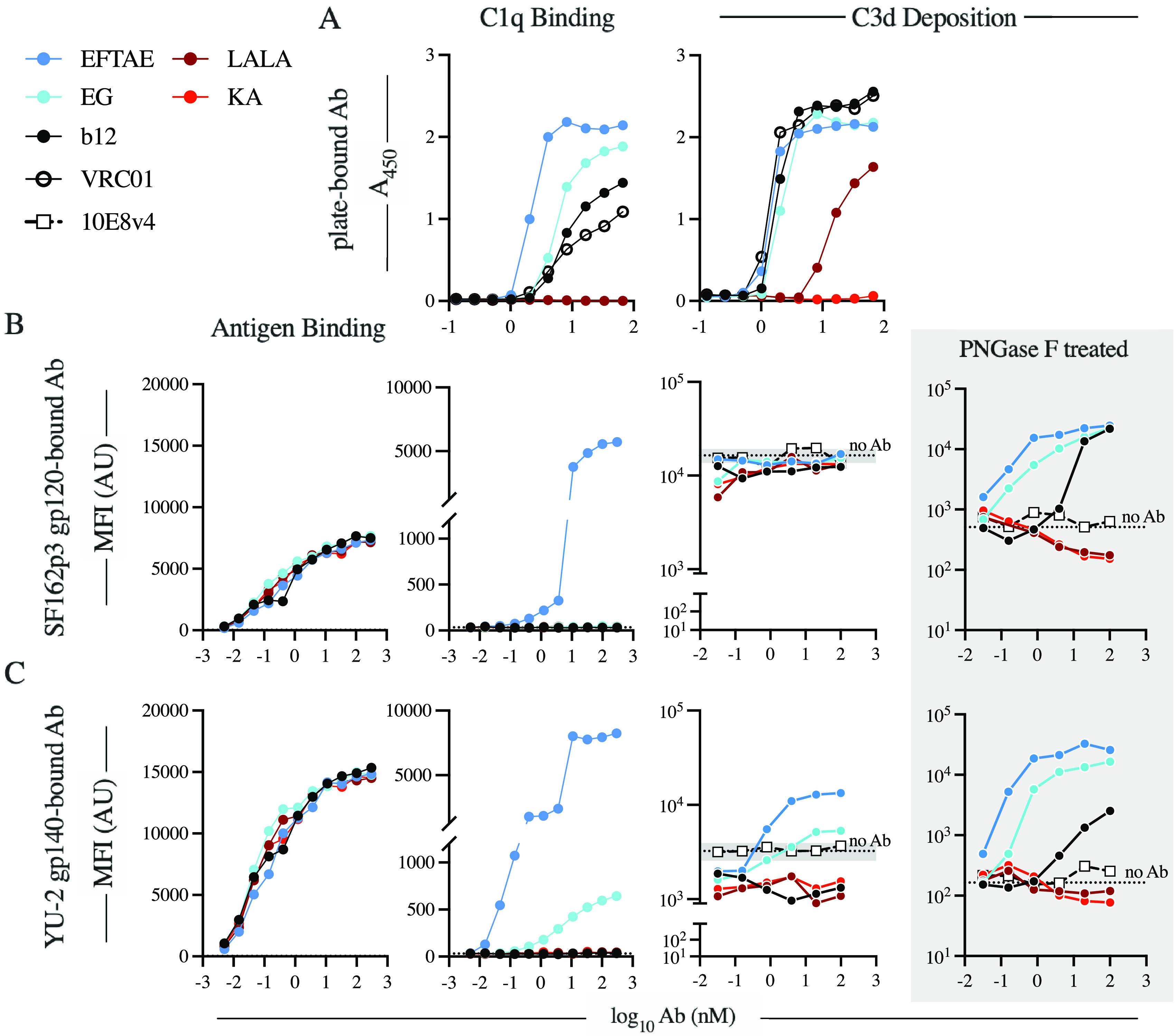
The ability of b12 to activate complement is influenced by assay setup and antigen context. (A) The antigen-independent ability of the antibody panel to bind C1q (left) and fix complement (right) was determined by ELISAs via antibody-coated wells. (B and C) Beads conjugated with SHIV_SF162P3_ gp120 (B) or HIV-1_YU-2_ gp140 trimer (C) were used to assay antigen binding (left), C1q binding (center), and complement fragment C3d deposition (right). Antigen beads treated with PNGase F were used to assess the impact of *N*-linked antigen glycosylation antibody-independent activation and to isolate antibody-dependent C3d deposition (shaded). For C3d deposition on non-PNGase F-treated antigen beads, background activity is reported as the average MFI (dotted line) ± standard deviation (shaded region on the *y* axis) of anti-C3d detected on beads in replicate wells of pooled NHS (*n* = 6) in the absence of antibody. Data are representative of results from two independent experiments. AU, arbitrary units.

To assess the inherent ability of each variant to engage C1 and fix complement, enzyme-linked immunosorbent assays (ELISAs) were performed using antibody-coated microtiter plate wells as described previously by Hessell et al. (10) (Fig. 2A). Variants LALA and KA showed ablated C1q binding relative to unmodified b12 and the isotype control VRC01 (Fig. 2A, left). Elevated C1q binding was observed for both the EFTAE and EG variants, which directly or indirectly improve the interaction of Fc with hexavalent C1q via improved C1q binding affinity or increased oligomerization potential, respectively. Detection of C3d was used as a measure of total C3 fixation as it is detectable regardless of postdeposition proteolytic processing. Deposition of C3d was undetectable for KA and dramatically reduced by the LALA mutations (Fig. 2A, right). Despite the greater C1q binding with the EG and EFTAE variants than that mediated by unmodified b12, unmodified and enhanced variants displayed equivalent levels of C3d deposition. Additionally, a moderate level of C3d deposition was retained by the LALA variant despite undetectable C1q binding.

### Complement activation is context dependent.

Structural studies have shown that C1q engagement depends heavily on the quaternary organization of adjacent antibody Fc domains (8, 20), which in turn can be influenced by the local concentration and orientation of antigen (21). Therefore, we sought to reevaluate C1q recruitment and C3d deposition within the context of antigen-antibody complexes.

Briefly, antibodies were first incubated with antigen-coupled microspheres before being assessed for C1q recruitment and C3d deposition. All Fc variants of b12 displayed similar levels of binding to the simian-human immunodeficiency virus SHIV_SF162P3_ gp120 monomer (Fig. 2B, left) and the HIV-1_YU-2_ gp140 trimer (Fig. 2C, left). In contrast to the ELISA results, however, unmodified b12 failed to recruit human C1q in the antigen-dependent assay (Fig. 2B and C, center). Only the EFTAE variant exhibited robust, concentration-dependent C1q engagement with both gp120 and gp140 antigens (Fig. 2B and C, center). In contrast, b12 EG complexed with the gp140 trimer (Fig. 2C, center) mediated detectable C1q recruitment but failed to do so in complex with the gp120 monomer (Fig. 2B, center). Antibody-dependent C3d deposition was not observed with SHIV_SF162P3_ gp120 (Fig. 2B, right) as activity was obscured by an elevated baseline level of complement fixation. Since antibody-dependent activity was resolved following treatment of antigen beads with peptide-*N*-glycosidase F (PNGase F) (Fig. 2B, shaded), the elevated background deposition can be attributed to lectin and alternative pathway activation by direct interaction with antigen glycosylation. In contrast, EFTAE and EG drove elevated C3d deposition when bound to the HIV-1_YU-2_ gp140 trimer, while those that did not recruit C1q, such as IgG1 b12, appeared to inhibit non-antibody-mediated activation pathways (Fig. 2C, right). Again, antibody-dependent differences in activation were further resolved using PNGase F-treated beads (Fig. 2C, shaded).

### Complement is activated by a clinically relevant anti-HIV-1 bnAb.

**FIG 3:**
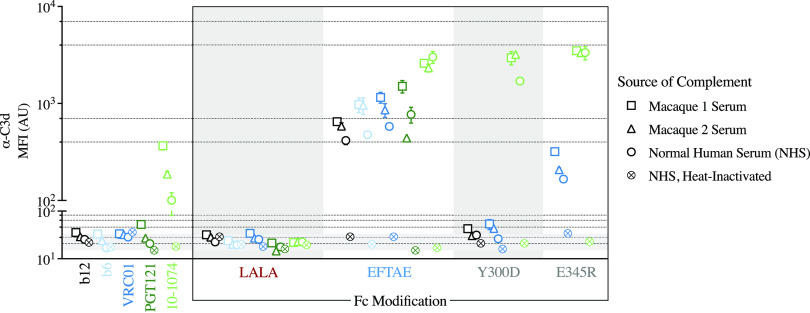
Complement is activated by the clinically investigated anti-HIV-1 bnAb 10-1074. The degree to which a panel of anti-HIV-1 antibodies fixes complement (C3d) on the surface of HIV-1_SF162_ gp140 trimer-conjugated beads was evaluated using both the complement-preserved serum of individual monkey donors (*n* = 2) as well as complement-active and heat-inactivated pooled human serum. The leftmost panel compares the activities elicited by antibodies with unmodified Fc domains, while the box to the right displays the same panel of antibodies, or a subset thereof, grouped by Fc variant identity, with color-coded points indicating the antibody specificity (black and blue indicate the CD4bs, and shades of green indicate the V3-glycan patch; light and dark shades indicate lower to higher relative neutralization potencies, respectively). Points and error bars represent the means and ranges from two technical replicates, respectively, with the shape indicating the source of complement assayed. Background fixation, reported as the average MFI of anti-C3d detected in replicate wells of pooled NHS in the absence of antibody, is indicated by a dotted line, with the range denoted by the shaded region on the *y* axis.

Multiple anti-HIV-1 bnAbs are currently being evaluated as agents of preexposure prophylaxis. In order to assess whether anti-HIV-1 antibodies undergoing clinical development are capable of driving complement activation and fixation, a small panel consisting of bnAbs (and a nonneutralizing monoclonal antibody [mAb]) targeting two distinct epitope regions was generated and evaluated for the ability to drive the deposition of C3 on Env-conjugated beads (Fig. 3). The panel consisted of the CD4 binding site (CD4bs)-specific bnAbs b12 and VRC01, the nonneutralizing anti-HIV-1 antibody b6, as well as the V3-loop-associated N332 glycan patch-specific bnAbs PGT121 and 10-1074. Additionally, Fc variants of these antibodies were produced to potentiate and knock out complement activation, with the goal of revealing antibody-specific differences. The Fc variants that were evaluated consisted of EFTAE, LALA, as well as the additional enhancing variants Y300D (20) and E345R (8, 16).

To begin to compare activities in human and rhesus macaques, the activity of pooled human serum with and without heat inactivation was compared to the activity observed in the presence of serum from two individual macaques. While higher levels of C3d deposition were generally observed for the macaque serum samples, variation between individuals appeared to be comparable to variation between species (Fig. 3). Significantly, trends between antibodies and Fc variants were consistent across different complement sources.

Of the unmodified anti-HIV-1 mAbs tested, only 10-1074 displayed detectable C3d deposition in each of the three sources of complement-preserved serum tested, and in each of the Fc-enhanced forms, 10-1074 displayed a nearly equivalently high anti-C3d signal (Fig. 3). The bnAb b12 drove fixation only in the EFTAE Fc format; however, the signal was moderate to low compared to those of the other EFTAE-modified mAbs tested. Interestingly, the point mutation E345R but not Y300D potentiated complement activation by VRC01. Such an observation might be attributable to antibody-specific differences in complement biology; the integration of these mutations with differences in the approach angle, orientation, or flexibility of the Fc ultimately may impact the propensity for Fc-Fc oligomerization and Fc-C1q association. Of note, the EFTAE variant uniformly potentiated complement activation across the bnAbs and nonneutralizing mAb tested, which is consistent with the proposed mechanism of directly increasing the affinity of the collagenous heads of C1q for IgG Fc.

### Increasing biological complexity modulates antibody-dependent complement activation.

**FIG 4:**
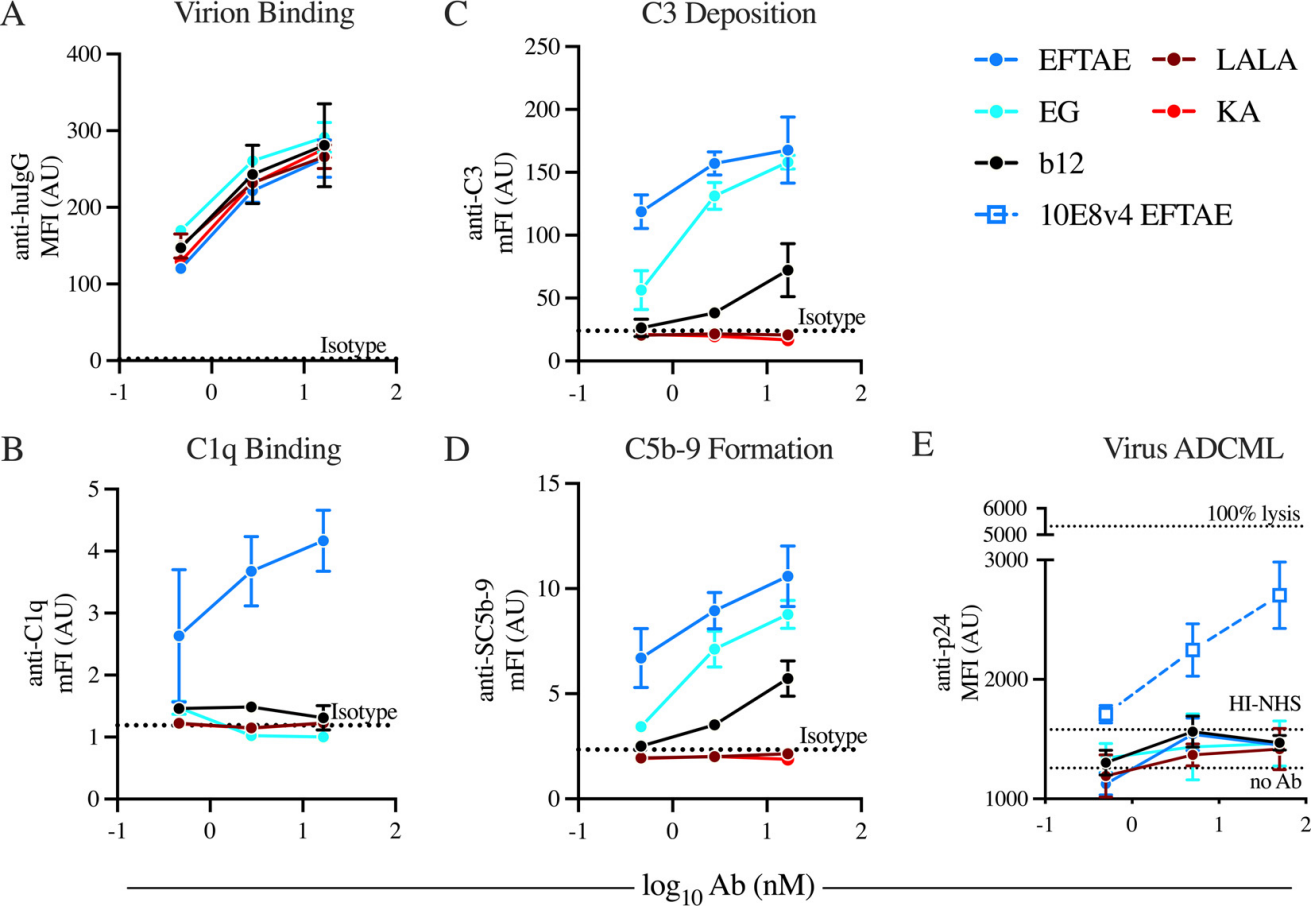
Complement-enhanced Fc variants of IgG1 b12 do not mediate viral lysis despite detectable deposition activity on viral particles. Antibodies were assayed for their ability to bind to the surface of HIV-1_BaL_ particles bound to lectin-conjugated magnetic beads (A), recruit C1q to the viral surface (B), and affect terminal complement activities, including C3 deposition (C), C5b-9 complex formation (D), and MAC-mediated lysis (E), determined by the detection of released capsid protein p24. Dotted lines represent average baseline complement deposition on beads in wells containing a nonspecific isotype control (A to D) or antibody-independent baseline complement deposition and heat-inactivated NHS (E). Points and error bars represent means ± standard deviations from technical triplicates, respectively. Data are reported as mean or median fluorescence intensities (mFI or MFI, respectively) and are representative of results from two independent experiments. huIgG, human IgG.

**FIG 5:**
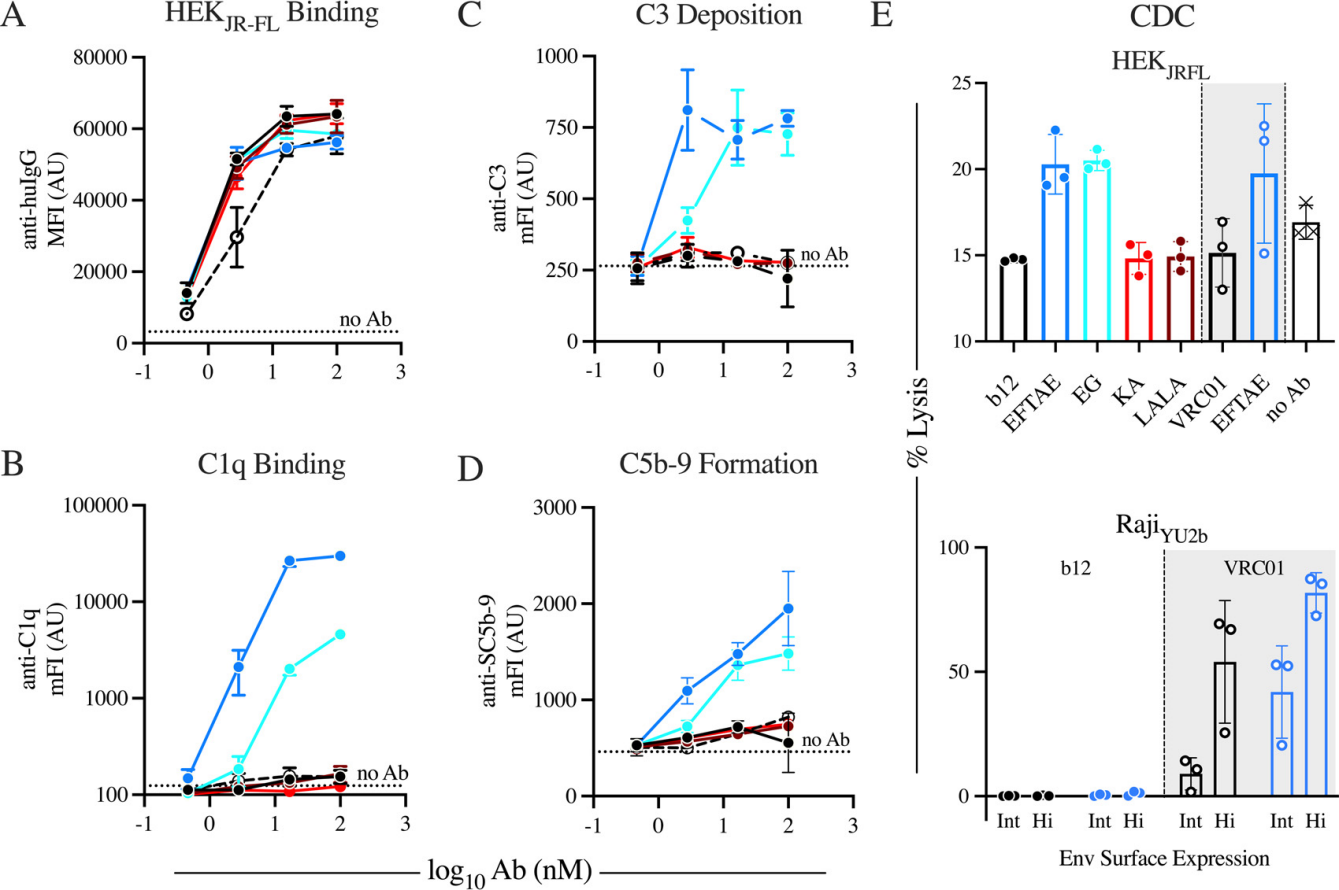
IgG1 b12 does not measurably direct complement activity against Env-expressing cells, while the CDC activity of enhanced Fc variants depends on the target cell. Antibodies were assayed for their abilities to bind to the surface of HIV-1_JR-FL_ gp140 transiently expressed on HEK293F cells (A), recruit C1q to HEK_JR-FL_ cells (B), and affect terminal complement activities targeting the antibody-opsonized HEK_JR-FL_ cell surface, including C3 deposition (C), C5b-9 complex formation (D), and MAC-mediated lysis of two distinct Env-expressing cell models (E). Dotted lines represent average baseline complement deposition on cells in wells lacking antibody (A to D). Points and error bars represent means from technical triplicates (A to D and E, top) or from three serum donors (E, bottom) and standard deviations, respectively. Data are representative of results from at least two independent experiments.

In the context of viral transmission, both virus and envelope on the surface of infected cells represent important targets of antibody-mediated prevention strategies. In order to approximate the behavior of b12 in more biologically relevant contexts and enable the evaluation of terminal complement activation, virus (Fig. 4) and envelope-expressing cells (Fig. 5) were used as target particles.

To evaluate antibody-driven complement activity against virus, lectin-conjugated beads were used to capture HIV-1_BaL_ virions and subsequently incubated with antibody and a source of complement. Antibody binding to lectin-bead-captured HIV-1_BaL_ (Fig. 4A) and the subsequent recruitment of C1q (Fig. 4B) revealed concentration-dependent virus binding for the antibodies tested and detectable recruitment of C1q by only the EFTAE Fc variant of b12, respectively. The complement cascade intermediate C3 (Fig. 4C) and C5b-9 (Fig. 4D), a precursor of membrane attack complex (MAC)-mediated lysis, were detected on lectin-conjugated-bead-captured HIV-1_BaL_ virions. Despite the limited detection of C1q, EFTAE and, to a lesser extent, EG were observed to drive both C3b deposition and C5b-9 membrane insertion, while unmodified IgG1 b12 displayed significantly weaker activity, and knockout variants reported signals equivalent to that of an isotype control. In contrast, virion lysis, as measured by the release of capsid protein p24, was not appreciably observed above background levels for any of the b12 variants tested, although antibody concentration-dependent complement-mediated lysis was observed with the EFTAE variant of the MPER-specific bnAb 10E8v4 (Fig. 4E).

To evaluate antibody-driven complement activity against cell-associated virus, HEK293F cells transiently expressing HIV-1_JR-FL_ gp140 (HEK_JR-FL_ cells) were incubated with antibody and a source of complement. While antibody binding to HEK_JR-FL_ cells revealed equivalent antigen binding (Fig. 5A), only the EFTAE Fc variant of b12 and, to a lesser extent, EG displayed detectable recruitment of C1q (Fig. 5B). In agreement with C1q binding, only the EFTAE and EG Fc variants of b12 promoted C3b deposition (Fig. 5C) and C5b-9 formation (Fig. 5D) on the surface of HEK_JR-FL_ cells, while unmodified forms of b12 and another CD4bs-specific antibody, VRC01, displayed a signal indistinguishable from those of complement knockout variants of b12 and control wells without antibody. In the HEK_JR-FL_ cell model, enhanced variants of b12 and VRC01 were observed to drive antibody-dependent complement-mediated cellular cytotoxicity, while unmodified and knockout versions appeared to protect cells from antibody-independent complement pathways (Fig. 5E, top). While enhanced b12 variants were shown to drive C3b deposition (Fig. 5C), C5b-9 complex formation (Fig. 5D), and complement-dependent cytotoxicity (CDC) (Fig. 5E, top) in the context of HEK cells transiently expressing HIV-1_JR-FL_ gp140, b12 in all formats tested failed to induce CDC of Raji cells stably expressing either intermediate or high levels of surface envelope (Fig. 5E, bottom). This deficiency was specific to b12, as CDC was robustly elicited by both unmodified and EFTAE forms of VRC01.

In summary, measures of sublytic complement activity detected on target virions (Fig. 3D and Fig. 4C) and envelope-expressing cells (Fig. 4D and Fig. 5C) mostly agreed with those detected on Env trimer-conjugated beads (Fig. 2C), at least in comparative antibody rank-order activity or activity interpreted as a binary variable. Distinctions between the detection of C1q recruitment (Fig. 4B) and complement deposition (Fig. 3D and Fig. 4C) were noted for unmodified and EG forms of b12. Importantly, unmodified b12 did not measurably direct lytic complement activity against either virus (Fig. 4E) or envelope-expressing target cells (Fig. 5E), although CDC mediated by complement-enhanced Fc variants highlights the differences that can exist between target cell models. Overall, these results demonstrate the degree of comparability between various measures of complement activity across several *in vitro* models and specifically indicate that IgG1 b12 is not an active driver of classical complement activation.

## DISCUSSION

Collectively, these data suggest that while unmodified and Fc variant forms of b12 exhibit the intrinsic biophysical interaction profile expected of them, they inherently lack the capacity to drive complement activity when biologically significant factors are incorporated into *in vitro* assays. Between intrinsic activity and that observed in the context of recombinant antigen, viral particles, and envelope-expressing cells used to model infected cells, we repeatedly observed differences in C1q binding, C3 deposition, C5b-9 formation, and lytic activity. Similarly, the identity (strain) and composition (monomer or trimer) (glycosylation state) of target antigens influenced diverse antibody-mediated complement phenotypes, and distinguishing antibody-mediated from antibody-independent complement activation was strongly dependent on the concentration of the complement source (data not shown). While these observations suggest that making predictions of *in vivo* activity from *in vitro* data appears not to be straightforward, the lack of activity observed for unmodified b12 across a variety of assay formats makes it clear that reinterpretation of the classic mechanism-of-action experiment is warranted. Importantly, this loss-of-function antibody infusion experiment definitively established a protective contribution of Fc receptor-mediated functions. However, because unmodified b12 appears not to exhibit meaningful complement-dependent activity in these and similar cell-based assays (22), its evaluation *in vivo* to inform on the contribution of complement to antibody-mediated prevention of infection appears to be limited.

Early studies implicated complement activation in HIV-1 infection enhancement (23–29). More recently, it has been reported that complement-opsonized virus promotes viral establishment in colorectal mucosa (30) and infection of mucosa-resident Langerhans cells (31). Evidence suggests that complement opsonization makes HIV-1 more accessible to host cells via interactions with complement receptors. In addition to mediating cellular adherence, complement-opsonized HIV was shown to be more efficiently internalized by (32) and overcome restriction in (33) dendritic cells (DCs). Downstream implications have been demonstrated to include interference with immature DC signaling pathways, resulting in decreased inflammatory and antiviral responses (34, 35) and reduced cytolytic potential of NK (34, 36) and CD8 T (30, 36) cells. Opsonization has also been hypothesized to contribute to viral reservoir maintenance in germinal centers via interaction with follicular dendritic cell-associated complement receptor 2 (CR2) (37). In line with this observation, hypofunctional CR2 polymorphisms have been shown to correlate with innate resistance to HIV-1 infection (38), and an association has been found between a single-nucleotide polymorphism (SNP) in the CR2 gene and susceptibility to infection among gp120-vaccinated individuals in a low-risk category (39).

While evidence suggests that upstream complement opsonins contribute to complement-enhanced infectivity, both HIV-1 virions and infected cells possess mechanisms for evading downstream complement-mediated lysis. Budding virions incorporate host membrane CD59 that inhibits complement fragment C9 polymerization and lytic pore formation (40). When this glycophosphatidylinositol (GPI)-anchored glycoprotein was blocked, complement-mediated viral and infected cell lysis was restored (41). However, the impact of CD59 on simian immunodeficiency virus (SIV) complement evasion was shown to be nearly negligible in experiments in which CD59 was cleaved from virion membranes with phosphoinositide phospholipase C (42). In addition to CD59 and other complement-restricting host factors (40, 43), the viral accessory proteins Vpu and Nef have been implicated in restricting membrane attack complex (MAC)-mediated lysis of infected primary CD4^+^ T cells by modulating surface Env levels (22).

Despite potential indications of complement-enhanced infection and the knowledge that pathogen and host factors facilitate HIV-1 complement evasion, studies that offer counterevidence exist, suggesting a possible antiviral role for antibody-driven complement activation. For example, plasma HIV-1 was shown to be susceptible to complement-mediated lysis (44), and plasma from both chronically and acutely infected patients triggered viral inactivation (45) and lysis (46) against an autologous virus isolate, with lytic activity being correlated with lower viral loads (46). These studies suggest that antibody-mediated complement-dependent virion lysis develops early in the course of infection and may reduce viremia *in vivo.* Also, lytic activity is not the only means by which complement may function. For example, an early study suggested that complement can neutralize plasma virus by a C5-independent mechanism (47), suggesting a possible mechanism of upstream complement component opsonization-mediated inactivation of HIV-1. A parallel observation has been made for another enveloped virus, human cytomegalovirus (HCMV), for which complement opsonization was demonstrated to enhance the *in vitro* neutralizing potency of monoclonal antibodies and immune sera (48). Furthermore, complement fragment opsonization may assist in the promotion of viremia-controlling adaptive responses (49–51).

Conflicting evidence for the role of antibody-mediated complement in the prevention or pathophysiology of HIV-1 may be explainable as being highly dependent on contextual variables such as HIV and host diversity as well as antibody-intrinsic factors. For example, in an early V3-specific antibody-mediated postexposure protection mouse study, artificially depleting the serum complement by treatment with cobra venom factor (CVF) was shown to abrogate the antibody’s protective capacity (52), with the caveat that while CVF treatment consumes nearly 100% of serum C3, large quantities of proinflammatory C3a and C5a, among other by-products, are generated in the process and represent a nontrivial systemic source of confounding factors. Also, V1V2-specific complement-activating serum IgG was defined as a correlate of reduced HIV-1 infection risk in the RV144 vaccine trial that demonstrated moderate protective efficacy (53).

Previous *in vitro* characterizations of monoclonal antibodies generated similar observations, that complement activities vary across distinct epitopes (54, 55), dependent on fine details of the geometry of antibody interactions with antigen (56, 57), C1q (8, 18, 58, 59), and self (oligomerization propensity) (8, 16). The collective result is that the activity observed in *in vitro* assays appears to be exquisitely sensitive to design details, challenging abstraction to expected effects *in vivo*. As examples, we and others have observed considerable differences between early (C3 deposition) and late (lytic) activities, between target cells or viruses, and in the context of antigen/virus diversity (22). Additionally, testing in nonhuman primates, and specifically in rhesus macaques, may introduce confounders to clinical translation related to as-yet-undiscovered cross-species differences in upstream factors influencing complement regulation or downstream processes like immune complex shuttling and processing or in relation to differences between HIV and SHIV. While interspecies differences may exist in the composition or the mechanistic or regulatory nuances of notably complex complement biochemistry, preliminary assessments comparing complement-preserved human and rhesus macaque sera resulted in relatively consistent levels of deposition on antibody-opsonized recombinant antigen beads. Moreover, the bnAb Fc variants maintained rank-order deposition signals independent of the species source of complement. While unresolved interspecies differences and well-established limitations of *in vitro* assessments constrain the scope of our findings, the data presented here call into question the perhaps widely held notion that complement is unimportant in antibody-mediated protection against HIV.

Given the suboptimal efficacy of antibody-mediated prevention of HIV-1 infection in its first field trial (7), means to improve diverse antiviral activities *in vivo* have renewed importance, and while b12 has been shown to lack the capacity to activate complement, we and others have observed that the V3-glycan patch-specific bnAb 10-1074 drives C3 deposition on Env-conjugated beads and Raji cells expressing Env (22), and the MPER-specific bnAb 10E8v4 drives lysis of HIV-1_BaL_ virions (D. A. Spencer, B. S. Goldberg, J. Dufloo, T. Bruel, S. Pandey, T. Cheever, P. Barnette, W. F. Sutton, H. Henderson, R. Agnor, L. Gao, O. Schwartz, N. L. Haigwood, M. E. Ackerman, and A. J. Hessell, unpublished data). Importantly, 10-1074 has been shown to suppress viremia in infected individuals (60) and, like several other bnAbs, is under clinical evaluation (ClinicalTrials.gov identifier NCT03554408) at the time of writing. While much remains unresolved regarding a comprehensive understanding of the role of complement-mediated activity in the antibody-based prevention or treatment of HIV-1 infection, the potential importance of either direct lytic functions or other antiviral or adjuvant-like activities should not be overlooked without further investigation. Indeed, complement-dependent lysis has been established as an important mechanism of action of the first monoclonal antibody therapeutic approved for the treatment of cancer, rituximab (61), which, unlike anti-CD20 antibodies targeting other epitopes (56), activates complement so strongly that it depletes serum complement during infusion (62). While our study and other studies of HIV-1 bnAbs in animal models suggest that the ability to use *in vitro* results to guide *in vivo* translation may be challenging, complement activity may yet prove to be a useful means to drive viral lysis and act in concert with direct neutralization and other effector functions or may alternatively play a role in pathogenesis. To this end, engineered antibody variants represent an important tool to understand the complex interplay among drug, host, and pathogen *in vivo* and thereby offer new means to improve upon our ability to prevent and treat HIV-1 infection.

## MATERIALS AND METHODS

### Generation of antibody Fc variants and recombinant antigens.

Plasmids for the soluble expression of the HIV-1_YU-2_ gp140 trimer (catalog number ARP-12133) and VRC01 heavy chain (catalog number ARP-12035) and light chain (catalog number ARP-12036) IgG1 were obtained through the NIH AIDS Reagent Program. DNA for b12 heavy and light chain sequences originate from Zwick et al. (63), DNA for b6 sequences originate from Roben et al. (64), and nucleotide sequences for PGT121 and 10-1074 were provided by Hugo Mouquet and Marina Caskey, and the cloning of these sequences was first reported in Mouquet et al. (65). Fc domain variants (EFTAE, EG, KA, LALA, Y300D, and E345R) of each antibody were cloned via site-directed mutagenesis using the QuikChange II site-directed mutagenesis kit (catalog number 200523; Agilent Technologies) according to the manufacturer’s directions. The desired incorporation of Fc substitutions was confirmed by Sanger sequencing (Genewiz).

Antibodies were produced using the Expi293 expression system. Briefly, heavy and light chain plasmids were transiently cotransfected into Expi293F cells (catalog number A14635; Thermo Fisher) according to the ExpiFectamine 293 transfection kit (catalog number A14525; Thermo Fisher) protocol. Culture supernatants were collected at 7 days posttransfection, purified by gravity using MabSelect protein A chromatography resin (catalog number 17519903; GE Healthcare), and polished via size exclusion chromatography (SEC) on a Superdex 200 column (catalog number 28989335; GE Healthcare).

The recombinant HIV-1_YU-2_ gp140 trimer was produced by transient transfection in HEK293F cells using polyethyleneimine (PEI) (catalog number 23966; Polysciences). Cell supernatants were harvested after 5 to 7 days, and clarified supernatants were purified by ion metal affinity chromatography with a HisTrap Excel column (catalog number 17-3712-06; GE Healthcare) on an Äkta pure chromatography system (GE Healthcare). Purified gp140 was polished via SEC on a Superdex 200 column (catalog number 28989335; GE Healthcare).

### Kinetics analysis of antibody binding to HIV-1 gp140 via biolayer interferometry.

The binding of unmodified b12 and Fc variants (KA, LALA, EG, and EFTAE) to the HIV-1_YU-2_ gp140 trimer was evaluated via BLI using the Octet RED96 instrument (Sartorius AG). Samples and controls were formulated in 1× kinetics buffer (1× phosphate-buffered saline [PBS], 0.1% bovine serum albumin [BSA], 0.02% Tween 20) and added to black 96-well F-bottom polypropylene microplates (catalog number 655209; Greiner Bio-One). High-precision streptavidin biosensors (catalog number 18-5117; Sartorius AG) were activated by soaking for 10 min in wells containing 5 μg/ml biotinylated anti-His tag antibody (catalog number 4603-08; Southern Biotech), followed by loading of the HIV-1_YU-2_ gp140 trimer (His tagged) to a threshold RU (response units) of 0.3 nm. After 60 s at baseline in 1× kinetics buffer, gp140-loaded biosensors were dipped into serially diluted IgG (0.171 to 125 nM) to measure association for 300 s, followed by dissociation for 300 s in kinetics buffer. A reference biosensor dipped into a well containing kinetics buffer was used to subtract nonspecific signal. Biosensors were regenerated to the anti-His tag antibody between fresh antigen loading and subsequent sample association and dissociation steps using regeneration buffer (10 mM glycine, pH 1.7). Kinetic constants were determined by fitting traces to a 1:1 binding isotherm by FortéBio HT analysis software (version 11.1.1.39).

### Antibody and Fc receptor binding in a microsphere-based immunoassay.

HIV-1_YU-2_ gp140 trimer and SHIV_SF162P3_ gp120 (catalog number IT-001-146p; Immune Technology Corp.) antigens were covalently coupled to coded MagPlex superparamagnetic carboxylated magnetic microparticles (Luminex Corp.) using carbodiimide cross-linking chemistry as previously described (66). To evaluate antibody binding by unmodified IgG and Fc variants, gp140- and gp120-coupled beads were incubated in a multiplex format with IgG for 1 h at room temperature (RT) with shaking. Plates were washed five times on an automated plate washer (catalog number 405; BioTek) and subsequently detected with 0.7 μg/ml phycoerythrin (PE)-conjugated goat anti-human IgG Fc (catalog number 2048-09; Southern Biotech) for 1 h at RT with shaking. Following five washes, beads were resuspended in xMAP sheath fluid (Luminex Corp.), and sample median fluorescence intensities (MFIs) were collected using the Magpix system (Luminex Corp.).

Human Fc receptor binding profiles were generated by a custom multiplex assay as previously described (66). Briefly, SHIV_SF162P3_ gp140-coupled beads were incubated with serial dilutions (50 pM to 500 nM) of IgG for 1 h at RT and washed five times using an automated plate washer. FcγR tetramers were prepared by incubating the biotinylated soluble human Fc receptors FcγRIIA, FcγRIIB, FcγRIIIA, and FcγRIIIB (generously provided by Shelly Krebs, U.S. Military HIV Research Program) with streptavidin-PE (catalog number PJRS25; Prozyme) in a 4:1 molar ratio for 20 min at RT. Detection reagent tetramers were incubated with antibody-antigen-bead complexes at 1.0 μg/ml for 1 h at RT with constant agitation. The excess tetramerized receptor was removed by five washes prior to flow cytometric analysis on the Flexmap 3D system (Luminex Corp.). Data are reported as the averages and ranges from two technical replicates. The background signal as a result of nonspecific interactions was minimal and not subtracted.

### Antigen-free C1q binding and C3d deposition determined by ELISAs.

Antibody-mediated complement activity was assessed in an antigen-independent manner through colorimetric ELISA-based measurements of C1q binding and C3d deposition. Antibodies were serially diluted 2-fold (0.13 to 66.67 nM) in coating buffer (50 mM carbonate-bicarbonate, pH 9.6) and immobilized to clear high-protein-binding-capacity 96-well ELISA plates (catalog number 423501; BioLegend) overnight at 4°C. Plates were subsequently washed three times with PBS-T (1× PBS, 0.05% [vol/vol] Tween 20) prior to blocking with 2% BSA for 2 h at RT.

For the detection of C1q binding, antibody-coated wells were incubated with 1 μg/ml biotinylated human C1q for 1 h at RT. Unbound C1q was removed by washing, and plates were subsequently incubated with a 1:200 dilution of horseradish peroxidase (HRP)-conjugated streptavidin (catalog number 890803; R&D Systems) for 20 min at RT. Next, plates were washed and developed with a TMB (3,3′,5,5′-tetramethylbenzidine) substrate solution (catalog number N301; Thermo Scientific) according to the manufacturer’s instructions. Human C1q (catalog number ab96363; Abcam) was biotinylated by primary amine coupling using EZ-Link sulfo-NHS (*N*-hydroxysuccinimide)-biotin (catalog number A39256; Thermo Scientific) according to the manufacturer’s instructions.

For the detection of C3d deposition, IgG-coated plates were incubated with normal human serum (NHS) (catalog number S174; Sigma-Aldrich) or heat-inactivated NHS (HI-NHS) (≥30 min at 56°C) diluted 1:100 in gelatin veronal buffer supplemented with Ca^2+^ and Mg^2+^ (GVB^++^) (catalog number G6514; Sigma-Aldrich) for 30 min at 37°C, placed on ice to halt the complement activation reaction, and washed three times with PBS-T. Next, a 1:1,000 dilution of biotinylated anti-human C3d (catalog number A702; Quidel) in PBS-T was added for 1 h at RT. After three washes, plates were incubated with streptavidin-HRP followed by detection with the TMB substrate as described above. Reactions were stopped with 0.16 M sulfuric acid, and absorbances were read at 450 nm on a SpectraMax Paradigm multimode UV-spec microplate reader (Molecular Devices). Wells that did not contain immobilized antibody served as background controls. Data shown are averages from two technical replicates and are representative of results from two independent experiments.

### C1q binding and C3d deposition using antigen-coated microspheres.

Antigen-coupled microspheres were prepared as described above and incubated in a multiplex format with IgG 3-fold serially diluted (5 pM to 300 nM) in assay buffer (1× PBS, 0.1% BSA, 0.05% Tween 20) for 1 h at RT. Following the removal of excess IgG with five washes in assay buffer, antibody-antigen complexes were assessed for their ability to recruit C1q and drive classical complement activation, as follows.

Assessment of C1q binding was carried out as previously described (66). Briefly, 1 μg/ml biotinylated human C1q was incubated for 1 h at RT, followed by washing and incubation with a 1:500 dilution of streptavidin-PE (catalog number PJRS25; Prozyme) for 30 min at RT. Beads were washed and resuspended in xMAP sheath fluid (Luminex Corp.) before acquisition on the Magpix system (Luminex Corp.).

For C3d deposition assays, NHS (catalog number S174; Sigma-Aldrich) diluted 1:100 in GVB^++^ was incubated with antibody-complexed antigen beads for 30 min at 37°C, placed on ice to stop complement activation reactions, washed, and incubated with 0.1 μg/ml biotinylated anti-human C3d (catalog number A702; Quidel) for 1 h at RT with shaking. Following washes, bound anti-C3d antibodies were detected by incubation with 1 μg/ml streptavidin-PE (catalog number PJRS25; Prozyme) for 20 min at RT, washed, and analyzed on the xMAP system (Luminex Corp.). For the experiment comparing the small panel of anti-HIV-1 antibodies, antibodies were assayed at 100 nM. Complement-preserved individual monkey serum was provided by the Oregon National Primate Research Center at Oregon Health & Science University, and NHS was sourced from a distinct vendor (catalog number A112; Quidel). To assess the impact of viral antigen *N*-linked glycosylation on antibody-independent activation, antigen beads were treated with PNGase F (catalog number P0704S; New England BioLabs) under nondenaturing conditions according to the manufacturer’s protocol. Briefly, antigen beads were mixed with GlycoBuffer 2 (10×), deionized water to a total volume of 20 μl, and PNGase F amidase; incubated at 37°C for 20 h; washed three times with PBS-TBN (TBN is 0.05% Tween 20, 1.0% BSA, 0.1% NaN_3_ [sodium azide]) (catalog number P0210; Teknova); resuspended in GVB^++^; and used immediately in the C3d deposition assay as described above.

The data shown are representative of results from at least two independent experiments. Assay wells containing assay buffer in lieu of antibody were used to assess background C1q association or complement activation driven by antibody-independent pathways. To control for nonspecific antibody-mediated activity, the anti-HIV-1 gp41 mAb 10E8v4 was used as a negative control in the gp120-conjugated bead experiments.

### Bead capture of viral particles.

To generate bead-captured HIV virions, aldrithiol (AT-2)-treated HIV-1_BaL_ (generously provided by Jeffrey Lifson, National Cancer Institute) was captured by lectin-coated beads via binding to glycan motifs on the viral surface. To prepare lectin-coated beads, 0.2 mg streptavidin Dynabeads (catalog number 11205D; Thermo Fisher Scientific) were washed five times with PBS and resuspended in 200 μl of biotinylated Galanthus nivalis lectin (catalog number B-1245-2; Vector Laboratories) diluted to a concentration of 0.1 mg/ml in PBS. The bead-lectin mixture was incubated for 20 min at RT with end-over-end rotation, followed by five PBS washes. Subsequently, 2 μg p24 of AT-2-treated HIV-1_BaL_ was incubated with 40 μg of lectin-coated beads for 90 min at RT with end-over-end mixing, washed three times using a magnet, and resuspended in PBS-BSA (1× PBS, 0.1% [wt/vol] BSA) for storage at 4°C.

The functional capture of virions and their ability to interact with anti-HIV-1 antibodies were tested by incubation with titrating amounts of anti-HIV-1 antibodies, followed by secondary staining with PE-conjugated goat anti-human IgG (catalog number 2040-09; Southern Biotech). After confirmation of homogeneous capture, bead-captured virus was subsequently used for C1q binding and complement deposition experiments. While the capture of glycosylated viral debris, including free, degraded, or monomeric components of Env, cannot be precluded from influencing the detection of C1q association and C3 deposition, the detection of C5b-9 can be viewed as validating viral capture as the stable insertion and polymerization of C6-9 require an intact lipid bilayer. To help ensure the structural integrity of viral particles, bead-captured virions were prepared fresh prior to each experiment.

### C1q binding, C3 deposition, and C5b-9 formation on bead-captured HIV virions.

To assess the ability of antibody-opsonized cell-free HIV to recruit C1q and drive complement activation, bead-captured virus was incubated with serially diluted IgG in GVB^++^ for 20 min at 37°C with gentle shaking. Concurrently, 1:10-diluted NHS in GVB^++^ was separately equilibrated to 37°C for 15 min. Following precomplexing of IgG with bead-captured virus, warmed NHS was directly added to the antibody-virus solution to achieve a final serum dilution of 1:20 and incubated for 1 h at 37°C with gentle shaking. To measure baseline complement activation, bead-captured virus was incubated with NHS in the absence of antibody. Excess antibody and serum were removed by two washes with PBS-BSA.

For the detection of C1q binding, serum-incubated bead-captured virus was incubated with mouse anti-human C1q (clone JL-1, catalog number HM2382; Hycult Biotech) at 3 μg/ml for 30 min on ice, followed by two PBS-BSA washes. Secondary staining was performed using anti-mouse IgG-Alexa Fluor 647 (AF647) (catalog number A-21235; Invitrogen) diluted to 1:1,000 in PBS-BSA. Complement activation was assessed by the detection of C3 deposition and C5b-9 formation. To measure the degree to which test antibodies mediated C3 deposition, serum-incubated bead-captured virus was incubated with fluorescein isothiocyanate (FITC)-conjugated anti-human C3 (catalog number C7652F; Cedarlane Labs) at 1 μg/ml for 20 min on ice, washed twice with PBS-BSA, and measured by flow cytometry as described below. To measure C5b-9 deposition, serum-incubated bead-captured virus was incubated with mouse anti-SC5b-9/terminal complement complex (TCC)/neoantigen (catalog number A239; Quidel) at 1 μg/ml, followed by two PBS-BSA washes and secondary staining with 1:200-diluted goat anti-mouse IgG-AF647 (catalog number A-21235; Invitrogen), and for the detection of antibody binding, 1 μg/ml PE-conjugated goat anti-human IgG (catalog number 2040-09; Southern Biotech) was used. Beads were washed out of the detection reagent and resuspended in PBS-BSA for flow cytometric analysis on a MACSQuant Analyzer 10 system (Miltenyi Biotec). Bead populations were first gated for singlets before determining the mean fluorescence intensity (mFI). Data are reported as means and standard deviations from three technical replicates and are representative of results from at least two independent experiments.

### Antibody-dependent complement-mediated virolysis assay.

Complement lysis of HIV-1 virions was assessed by measurement of capsid protein p24, released following viral membrane disruption. In 96-well polystyrene tissue culture-treated microplates (catalog number 6916A05; Corning), 0.38 ng p24 of AT-2 HIV-1_BaL_ and a 1:50 dilution of human complement serum (catalog number S1764; Sigma-Aldrich) were mixed with IgG in GVB^++^ for a total volume of 150 μl. To generate a p24 standard curve, disruption buffer (catalog number 5421; ABL, Inc.) was added at a 1:10 dilution to serially diluted virions. Heat-inactivated complement serum (56°C for 30 min) and wells containing active complement serum without antibody served as negative controls for baseline p24 concentrations and complement-mediated lysis via antibody-independent pathways, respectively. Plates were incubated at 37°C for 1.5 h with gentle shaking before transferring 80 μl to black 96-well clear flat-bottom plates (catalog number 655906; Greiner Bio-One). Each sample was assayed in triplicate.

Quantification of released p24 was carried out using a bead-based sandwich assay. Briefly, MagPlex beads conjugated with two monoclonal murine anti-p24 antibodies (catalog numbers ab9072 and ab9044; Abcam) were incubated with each sample for 1 h at RT with gentle orbital shaking (600 rpm), followed by five washes on an automated plate washer. p24-bound beads were detected via 0.5 μg/ml polyclonal rabbit anti-p24 antibodies (catalog number NBP2-41214; Novus Biologicals) for 1 h at room temperature with shaking, washed five times, and stained with 0.6 μg/ml R-PE-conjugated rat anti-rabbit Ig (catalog number 4065-09; Southern Biotech). After incubation and washing steps, beads were resuspended in xMAP sheath fluid (Luminex Corp.), and MFI values were recorded by the Magpix system (Luminex Corp.). Data are reported as means and standard deviations from three technical replicates and are representative of results from at least two independent experiments.

### C1q binding, C3 deposition, and SC5b-9 formation on cell surface-expressed gp140.

FreeStyle HEK293F cells (catalog number R79007; Thermo Fisher Scientific) were transiently transfected with a plasmid encoding HIV-1_JR-FL_ gp140 C-terminally linked to a GPI anchor using PEI (Polysciences). After 48 h, cells were washed out of culture media, and 1 × 10^5^ live cells were transferred to each well of a 96-well cell culture plate. Fivefold serially diluted IgG (0.8 to 100 nM) was mixed with NHS or HI-NHS diluted in GVB^++^ (for a final dilution of 1:20) and incubated with gp140-expressing cells for 1.5 h at 37°C with gentle shaking. Following incubation and prior to detection of C1q recruitment and two signatures of complement activation, samples were transferred to 96-well V-bottom plates and washed three times with cold PBS-BSA.

For the detection of C1q binding, cells were incubated with mouse anti-C1q (clone JL-1, catalog number HM2382; Hycult Biotech) at 5 μg/ml and washed, and secondary staining was performed with 1:1,000-diluted anti-mouse IgG-Alexa Fluor 647 (catalog number A-21235; Invitrogen). To measure levels of C3 deposition, cells were stained with a secondary detection solution consisting of 1 μg/ml FITC-conjugated mouse anti-human C3 (catalog number CL7632F; Cedarlane Lab), 1 μg/ml PE-conjugated goat anti-human IgG (catalog number 2040-09; Southern Biotech), and 2 μg/ml propidium iodide (catalog number P1304MP; Thermo Fisher) for 30 min on ice. For C5b-9 formation, mouse anti-SC5b-9 (catalog number A239; Quidel) was incubated at 1 μg/ml, followed by two washes and secondary staining with 1:200-diluted goat anti-mouse IgG-AF647 (catalog number A-21235; Invitrogen), 1 μg/ml PE-conjugated goat anti-human IgG (catalog number 2040-09; Southern Biotech), and 2 μg/ml propidium iodide.

Cells were washed out of the detection reagent and resuspended in PBS-BSA for flow cytometric analysis on a MACSQuant Analyzer 10 system (Miltenyi Biotec). The degrees of C1q binding, C3 deposition, and C5b-9 formation are shown as mean fluorescence intensities of the singlet population gated on live gp140-expressing cells. Baseline antibody-independent levels of C1q, C3 deposition, and C5b-9 formation were measured by incubating cells with NHS in the absence of antibody. Data are reported as means and standard deviations from three technical replicates and are representative of results from at least two independent experiments.

### Transiently expressed HIV-1_JR-FL_ gp140 HEK complement-dependent cytotoxicity assay.

FreeStyle HEK293F cells were transiently transfected to express HIV-1_JR-FL_ gp140 as described above. In 96-well U-bottom tissue culture plates, antibodies and NHS were added to 2 × 10^5^ live cells per well to achieve final concentrations of 20% (vol/vol) NHS and 100 nM IgG in GVB^++^. Negative-control wells without antibody were included to determine baseline antibody-independent complement activation. Plates were incubated for 4 h at 37°C with orbital shaking before washing three times with PBS. Cells were stained with the live/dead fixable aqua dead cell marker (catalog number L34957; Life Technologies) diluted 1:250 in PBS and incubated for 30 min on ice. Wells were subsequently washed twice with PBS containing 1% BSA before resuspending the cells in PBS-BSA for analysis on the flow cytometer. Percent lysis was calculated as the number of cells staining strongly positive for the aqua dead cell marker divided by the total number of cells processed. Data are reported as means and standard deviations from three technical replicates and are representative of results from at least two independent experiments.

### Env^+^ Raji cell complement-dependent cytotoxicity assay.

Complement-dependent cytotoxicity (CDC) of Raji cells transduced with a retroviral vector encoding HIV-1_YU-2b_ Env was assessed as previously described (22). Briefly, transduced cells were generated by sorting for expression via a green fluorescent protein (GFP) reporter signal and clonally expanded to obtain cells expressing intermediate or high *env* levels. Cells were mixed with 50% NHS or 50% heat-inactivated human serum and 10 μM IgG for 24 h at 37°C. Complement-mediated lysis was measured with the live/dead fixable aqua dead cell marker (catalog number L34957; Life Technologies) prior to fixation and flow cytometric analysis (Attune NxT; Invitrogen). CDC was reported as the relative percentage of dead cells compared to a “no-antibody” condition. Biological replicates consisted of three independent serum donors. Rituximab was used as a positive control for strong antibody-mediated CDC.

### Data analysis.

Structural images were rendered with PyMOL (Schrodinger). Graphical plots and analyses were performed in GraphPad Prism (version 9.0).

### Data availability.

The data sets generated and/or analyzed during the current study are available from the corresponding author upon reasonable request.
